# Non-genomic action of beclomethasone dipropionate on bronchoconstriction caused by leukotriene C_4_ in precision cut lung slices in the horse

**DOI:** 10.1186/1746-6148-8-160

**Published:** 2012-09-10

**Authors:** Maria Fugazzola, Ann-Kristin Barton, Frank Niedorf, Manfred Kietzmann, Bernhard Ohnesorge

**Affiliations:** 1 University of Veterinary Sciences Hanover, Clinic for Horses, Hanover, Germany; 2 University of Veterinary Sciences Hanover, Institute for Pharmacology, Pharmacy and Toxicology, Hanover, Germany

**Keywords:** Beclomethasone, Horse, Leukotriene, Non-genomic membrane receptors, Recurrent airway obstruction, RAO, Precision-cut lung slices, PCLS

## Abstract

**Background:**

Glucocorticoids have been proven to be effective in the therapy of recurrent airway obstruction (RAO) in horses via systemic as well as local (inhalative) administration. Elective analysis of the effects of this drug on bronchoconstriction in viable lung tissue offers an insight into the mechanism of action of the inflammatory cascade occurring during RAO which is still unclear. The mechanism of action of steroids in treatment of RAO is thought to be induced through classical genomic pathways. We aimed at electively studying the effects of the glucocorticoid beclomethasone dipropionate on equine precision-cut lung slices (PCLS).

PCLS were used to analyze ex-vivo effects of beclomethasone on inhibiting bronchoconstriction in the horse. The inhibiting effect was measured through instillation of a known mediator of inflammation and bronchoconstriction, leukotriene C_4_. For this, the accessory lobes of 13 horses subjected to euthanasia for reasons unrelated to the respiratory apparatus were used to obtain viable lung slices.

**Results:**

After 30 minutes of PCLS incubation, beclomethasone showed to significantly inhibit the contraction of the bronchioles after instillation with leukotriene C_4_. The EC_50_ values of the two contraction curves (LTC_4_ with and without BDP) differed significantly from each other (p = 0.002). The possibility of a non-genomic rapid mechanism of action seems likely since transcriptional activities require a longer lag period.

**Conclusions:**

In human neuroendocrinology, high levels of glucocorticoids have been proven to function via a non-genomic mechanism of membrane receptors. The concentration of beclomethasone used on the lung slices in our study can be considered as high. This allows speculation about similar rapid non-genomic mechanisms of high-dosage inhaled glucocorticoids in the lower airways of horses. However, further assessment on a molecular basis is needed to confirm this.

## Background

The exact interactions of the single inflammatory mediators within the inflammatory cascade taking place during recurrent airway obstruction (RAO) in the horse are still unclear. Ex-vivo precision-cut lung slices (PCLS) from terminal airways of the horse offer a unique condition where the interaction of single inflammatory agents (i.e. bronchoconstrictors) and anti-inflammatory agents (i.e. bronchodilators) can be observed without multifactorial influence as occurs in vivo. This may be useful to further understand the pathogenesis of RAO. The aim of the study was to take a first step to analyse the interaction of two substances involved in bronchoconstriction and bronchodilation in healthy pulmonary tissue of 13 horses.

Synthetic glucocorticoids are recognized to be effective in the treatment of RAO in horses and have been widely used via various routes of administration in the past 15 years. Their beneficial effects are thought to be mainly achieved through their potent anti-inflammatory properties. They inactivate NF-kB, blocking transcription of genes coding for pro-inflammatory cytokines, up-regulate and increase the sensitivity of β_2_-adrenoceptors and reduce production of pro-inflammatory eicosanoids [1]. Their mechanism of action is mainly genomic with lag periods ranging from at least four to several hours or even days the manifest action of drug-induced response takes place [2-4].

Inhalation of glucocorticoids has become a good alternative to systemic administration in chronic obstructive airway diseases where high local concentration of the drug, lower dosage and the low risk of unwanted systemic side effects represent the major advantages of way of administration [5,6]. Beclomethasone dipropionate (BDP) is a widely administered inhaled corticosteroid in equine practice [7,8], although in vivo studies have shown that the marked improvement of clinical signs of airway obstruction after administration of low-dose inhaled beclomethasone was not accompanied by a decrease in airway inflammatory cells or a suppression of transcription factors NF-kB and AP-1 DNA-binding activity [9].

In the respiratory apparatus, the small airways are most affected by inflammatory processes of RAO [8,10,11] and represent the major target tissue for anti-inflammatory agents and bronchodilators.

In the past, leukotrienes have been shown to mimic many of the pathophysiological processes in allergic airway disease [12,13] and since leukotriene C_4_ is renowned for its potent bronchoconstricting action, it was chosen as bronchoconstrictor in our study.

Scientific investigations on the possible role of the cysteinyl leukotrienes in RAO have been reported in recent years. Lavoie et al. could not demonstrate improved lung function in horses with RAO after administration of a leukotriene D_4_ antagonist [14]. A more recent study suggested that there is increased leukotriene B_4_ and C_4_ production in airways of horses affected with RAO which may contribute to infiltration of neutrophils into the lungs and the sustained inflammation associated with RAO [15]. In an earlier study, the inhalative administration of LTD_4_ and LTB_4_ to healthy horses caused bronchoconstriction and neutrophil accumulation respectively [16]. Specifically, ex vivo LTC_4_ caused marked bronchoconstriction in equine PCLS which could be antagonized by the receptor antagonist MK-571 [17]. A hyper-reactivity of the bronchioles of RAO-affected horses in comparison to healthy horses was also demonstrated [17]. To date, no in vivo studies on effects of LTC_4_ have been reported.

We aimed at examining the effects of directly instilled beclomethasone on viable equine terminal airways to determine its capacity of inhibiting bronchoconstriction.

## Methods

### Animals

13 horses (8 geldings, 4 mares, 1 stallion; 16 ± 6 years of age [mean ± SD]; 500 ± 90 kg of body weight) which were to be euthanized due to conditions unrelated to this study or pulmonary disease were used for this study. After a general clinical examination, a specific examination of the respiratory apparatus consisting of auscultation, percussion, bronchoscopy, TBS-analysis and blood gas analysis was performed to exclude respiratory disease. Seven of the horses had received non-steroidal analgesics and antibiotic therapy related to the primary problem in the period preceding our study. After sedation (Detomidine, 0,02 mg/kg iv), euthanasia was performed using an overdose of pentobarbital.

### Experimental procedures

The method for production of PCLS was congruous with the one validated in previous studies [18-20] and will therefore be described summarily.

Immediately *post mortem,* the *accessory lobe* was removed with a cutting wire loop which was placed around the base of the lobe after opening the thorax and subsequently chilled to 4°C on ice, decreasing metabolic activity in the tissue without losing viability. In order to harden the lung tissue, the lobe was filled with a 1,5% low melting point agarose solution (type VII, Sigma-Aldrich Chemie, Germany) [21]. The agarose was enriched with cell culture medium (RPMI-1640 Medium, Biochrom AG Berlin, Germany) to provide nutrition to the tissue throughout the cutting procedures. Subsequently, precision cut lung slices with a diameter of 8 mm and a thickness of 400 μm were obtained from the lung tissue with a microtome (Krumdieck Tissue Slicer Model MD 4000, Alabama Research and Development). At the centre of each slice, the integrity, i.e. the presence of a terminal bronchiolus which was orientated perpendicular to the cut surface was condition of selection. After bedding the obtained PCLS in RPMI culture medium, viability was checked for each slice by contraction with metacholine (10^-5^ mol/l). A minimum of 50% lumen reduction was chosen as selection criterion. Viable slices were then washed with RPMI medium three times and incubated for 30 minutes to allow dilatation. Absence of pre-contraction was assessed by comparing initial values of bronchiolar lumen diameter with those after the reopening phase before the first contraction with leukotriene C_4_ was performed.

The concentration of LTC_4_ used for bronchoconstriction was based on an earlier study on PCLS where CysLT_1_ antagonists had been examined [17].

After checking quality and viability of the slices, LTC_4_ was applied to 6 PCLS of each horse to establish the individual reactivity of the different horses’ tissue. The concentration of the bronchoconstrictor was increased for each application (every two minutes), starting with 10^-15^ mol/l up to 10^-8^ mol/l. A visual example of this procedure is shown in the Additional file 1. The effects of the bronchoconstrictor LTC_4_ were recorded through digital photography two minutes after every increase of concentration. After rinsing, 3 PCLS were incubated for 30 minutes with BDP at the concentration of 10^-5^ mol/l and 3 PCLS remained untreated. After incubation and dilatation of all 6 PCLS, another contraction series with LTC_4_ was performed.

The effects of the bronchoconstrictor LTC_4_ with and without beclomethasone dipropionate were recorded through digital photography after every increase of concentration.

As a final step, the PCLS were again rinsed and incubated for 30 to 40 minutes in order to dilate. A further contraction with 10^-8^ mol/l LTC_4_ was performed to assure viability of the slices throughout the procedures. If a 50% lumen reduction was not achieved at this point, slices where excluded from statistical analysis.

The photographed lumen area of the bronchioles after each concentration increase was measured three times manually with a digitalising tablet (Trust®) and the average value was used. This value was quantified with percentage digital measurement (“Scion Image Version Beta 4.0.2”) and statistically analysed afterwards.

### Statistics

In order to compare reactivity of PCLS to LTC_4_ in our study to results of earlier studies, an EC_50_ was calculated. EC_50_ was defined as the concentration at which the PCLS contracted their lumen to 50% of the original surface area. Where a minimum of 50% lumen reduction was not reached, the EC_50_ concentration was defined as > −8 mmol/l. The effect of beclomethasone was compared with untreated control slices by comparing the EC_50_ values of the two groups using the Wilcoxon test. In order to obtain one data point per increasing LTC_4_ concentration, the average of 3 PCLS measurements per horse was used.

A p-value < 0.05 was regarded significant.

The software used for statistic evaluation was Graph Pad Prism 5.

## Results and discussion

**Figure 1:**
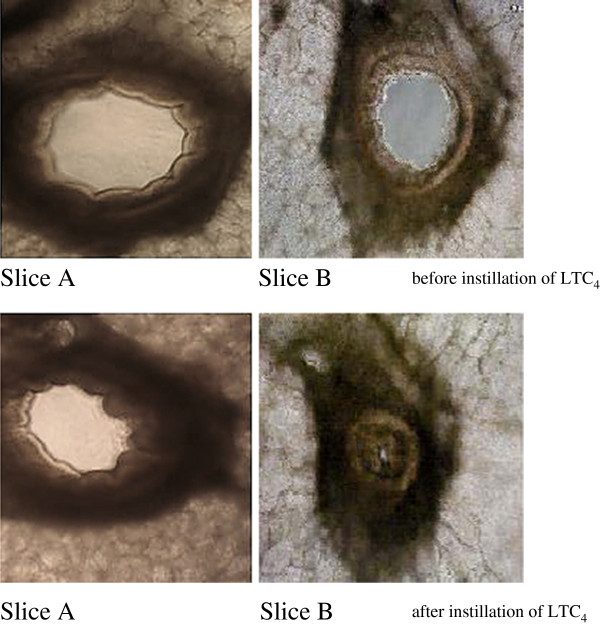
**Comparison between BDP-treated and untreated PCLS, and their reaction to instillation of LTC**_**4**_**(10**^**-8**^ **mol/l).** Top row: untreated slices (slice **A** and **B**). Bottom row: after instillation with LTC_4_, with slice **A** previously treated with BDP at 10^-5^ mol/l, slice **B** untreated. Magnification 100x (Zeiss® Axiostar Microscope Universal Digicam Adapter).

**Figure 2:**
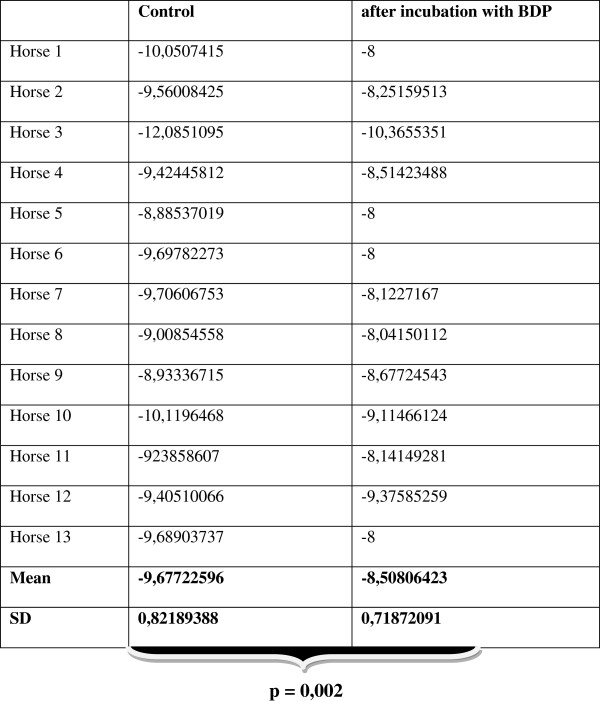
**Calculated EC**_**50**_**values for each horse before and after treatment with 10**^**-5**^ **mol/l beclomethasone dipropionate.** Means of the calculated EC_50_ values of the two contraction series (LTC_4_ with and without BDP) differed significantly from each other (p = 0.002). The number indicates the negative power of 10 (mol/l) as the expression of the LTC_4_ concentration needed to reach 50% of lumen reduction.

**Figure 3:**
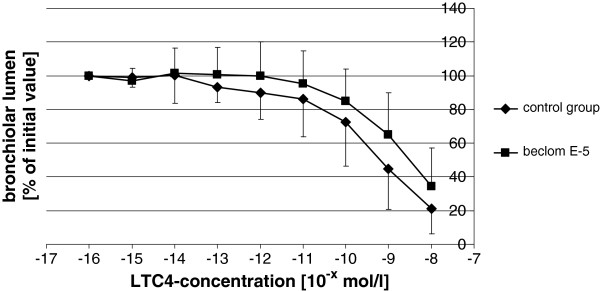
**Graphical representation of inhibitory effect of beclomethasone dipropionate on LTC**_**4**_**-induced bronchoconstriction.** Indication of the bronchial lumen (given in % of initial value) in dependency of LTC_4_ concentrations (given in 10^-x^ mol/l). Data shown are means ± SD of 13 horses.

From each *accessory lobe*, 40 to 60 viable slices could be obtained. After exposure to LTC_4_, the PCLS treated with BDP for 30 minutes showed less contraction than the untreated slices. As shown in Figure 1, at the applied concentration of 10^-5^ mol/l, BDP had a highly significant inhibitory effect on LTC_4_-induced bronchoconstriction. Whilst lumen reduction in the untreated PCLS started at a concentration of 10^-13^ mol/l LTC_4_, the beclomethasone group only started to react at a concentration of 10^-11^ mol/l LTC_4_. Moreover, the treated PCLS showed a lower degree of contraction than the untreated slices even at the highest concentration of LTC_4_. The calculated EC_50_ values of the two contraction series (LTC_4_ with and without BDP) differed significantly from each other (p = 0.002) (Figure 2). In the BDP-treated group a right shift of the concentration-effect curve, i.e. the increase of LTC_4_ concentration needed to induce a 50% bronchial lumen reduction, was obvious in PCLS of all but one horse when compared to the untreated group (Figure 3).

The calculated EC_50_ for LTC_4_ during the first contraction series of all included PCLS was 2 x 10^-10^ mol/l whilst the calculated EC_50_ for LTC_4_ after 30 minutes incubation with BDP (10^-5^ mol/l) was 3 x 10^-9^ mol/l.

The well-investigated genomic anti-inflammatory effect of glucocorticoids is mediated by their binding to a cytoplasmic receptor which, when activated, migrates to the nucleus. The activated receptor can interact with transcription factors (i.e. NF-kappaB, AP-1) inhibiting the synthesis of pro-inflammatory proteins (i.e. cytokines, COX-2). This trans-repressive activity explains most of the anti-inflammatory effects of glucocorticoids [22]. Since this mechanism is dependent on protein synthesis, the manifest action of the glucocorticoid takes place only after the characteristic lag period which ranges from at least four to several hours or even days [2-4,23].

In our study, incubation periods of 30 minutes with BDP seem too short for transcriptional procedures so it is likely that the inhibiting effect was mediated by a non-genomic mechanism of action which occurs instantaneously after exposure or within a very short latency period [2-4,24,25]. The non-genomic receptors directly activate various second messengers (e.g. Ca^++^-dependant protein kinases, cAMP) [26,27] which regulate specific biological actions within the cells such as bronchodilation, secretion, absorption etc. A controversy regarding the identity of receptors that mediate non-genomic, transcription-independent cellular responses to steroids is presently attracting considerable scientific interest [2,4,28,29]. There is strong evidence that classic receptors belonging to the nuclear receptor superfamily mediate non-genomic steroid effects in some cases [30]. In the human brain, the rapid non-genomic effect of corticoids seems to depend on classical mineralocorticoid receptors which are accessible from the outside of the plasma membrane and display a 10-fold lower affinity for corticosterone than the nuclear version involved in neuroprotection. Consequently, this type of receptor seems to play an important role while corticosteroid levels are high, i.e. during the initial phase of the stress response [31].

In our case, the concentration of BDP (10^-5^ mol/l) used for our study was based on Chanoine´s studies on rat lungs where intratracheal administration of the same concentration of BDP was used to assess pharmacokinetics [32]. Considering the short distance of diffusion and the small amount of lung tissue in PCLS treated with each application of BDP, 10^-5^ mol/l may be considered a high dosage. Since this dosage might be difficult to achieve in vivo, lower dosages should be assessed in following studies in order to confirm clinical relevance of these findings.

Another consideration is that a classic genomic mechanism would prevent bronchoconstriction by inhibiting at a higher level of the inflammatory signal cascade. Thus, final products such as leukotriene would not be produced. Since, in our study, the inflammatory mediator was directly instilled on the PCLS, a genomic mechanism of action could not occur; instead, a direct action of the glucocorticoid is most likely.

The pharmacological action of beclomethasone on LTC_4_ catabolism in transformed human bronchial epithelial cell lines accelerates the degradation of LTC_4_ into less active (less contractile) LTE_4_ and LTD_4_[33]. This mechanism is unlikely the same as in our study since increase of LTC_4_ catabolism was observed only after 2-day BDP incubation period of the cells, suggesting new protein synthesis.

In vivo BDP has proven to be effective in the therapy of RAO in horses [34,35]. The first signs of clinical improvement could only be seen after four days of treatment. Although this would indicate a complex trans-repressive mechanism of action, Couteil et al. could not show a decrease in pro-inflammatory transcription factors (NF-kappaB, AP-1) in the BALF despite clinical signs improving significantly [9]. These findings are in line with our results and suggest that clinical improvement (in our study inhibition of bronchoconstriction) was achieved through a fast-acting non-genomic mechanism.

## Conclusion

In this study, the mechanisms of action through which inhibition of bronchoconstriction was achieved were not investigated. There is evidence for a rapid and non-genomic reduction of intracellular [Ca2+] induced by aldosterone in human bronchial epithelium [36]. This could give reason to speculate about an inhibiting action of BDP on bronchoconstriction in PCLS through rapid calcium reduction in smooth muscle cells. In conclusion, we may say that further investigation is needed to understand the exact mechanism of action with which BDP at a high dosage inhibits bronchoconstriction in terminal airways through a non-genomic pathway.

## Abbreviations

BALF: Broncho-alveolar lavage fluid; BDP: Beclomethasone dipropionate; LTC: Leukotriene C; LTD: Leukotriene D; LTE: Leukotriene E; PCLS: Precision cut lung slices; RAO: Recurrent airway obstruction; TBS: Tracheobronchial secretion.

## Competing interests

We declare that we have no competing interests.

## Authors’ contributions

MF carried out the clinical and pharmacological studies, participated in the statistical analysis and drafted the manuscript. A-KB supervised part of the clinical and pharmacological studies. FN conceived the study in part and carried out the statistical analysis. MK contributed essential interpretation of data. BO conceived the study and contributed in interpretation of data. All authors read and approved the final manuscript.

## Supplementary Material

Additional file 1 **Contraction of bronchiolus after instillation of 10**^**-13**^**mol/l LTC**_**4**_**.** Click here for file
